# Diseases of the Fingers : B—Paronychia

**Published:** 1891-08-08

**Authors:** 


### SURGERY.

### DISEASES OF THE FINGERS (*continued*).

B.—Paronychia.

Under this term may be included all inflammatory affections involving the neighbourhood of the nail and its matrix. As before stated, the anatomical structures which may be affected are (1) the finger "pulp," which is a vascular and highly sensitive fibrous tissue, binding the skin firmly down to the periosteum of the ungual phalanx; (2) dense connective tissue at the sides of and behind the nail; and (3) the insertion of the extensor tendons and of the deep flexors by a broad inelastic expansion into the base of the terminal phalanx.

We have to deal with dense, vascular, highly sensitive tissues, exposed to constant changes of temperature and to innumerable minor accidents. Hence we can understand the frequency of inflammatory affections and their painful nature; also the great danger of allowing them to progress unchecked.

The commonest form of paronychia begins as a red swelling by the side of the nail, where the skin is thinner than elsewhere. There is frequently a history of a scratch or cut, and the patient is often engaged in some occupation which is injurious to the epidermis, such as washing clothes, handling soda, &c., &c. In such cases there is probably some dirt, containing germs, introduced through the weak or wounded epidermis. If care is taken of this, and warm fomentations applied, and the general health attended to, it is an affair of only a day or two, and usually ends by the formation of a pustule, or by gradual resolution.

If, however, the inflamed spot is neglected, and especially if the health is impaired, we get the next form of paronychia, which is a mild kind of whitlow. In this case the inflammation spreads slowly in the connective tissue, and suppuration results, but the tendinous structures escape, and the pus points, where the skin is thinnest, at the side of the finger or nail, which becomes loosened. It is quite impossible to say whether this inflammation is going to spread deeper still, so that every case should be treated promptly, viz., by hot fomentations, frequently changed, and if improvement does not occur quickly by free incision.

Gaucher has recommended the following plan:—Wet the inflamed part, and then rub over it a stick of solid nitrate of silver. He claims for this that all pain dies away, and the inflammation is arrested. In the early stages it is probable that this might be efficacious, the strong caustic soaking in and destroying the germs. It is no use when suppuration has set in.

*Paronychia Tendinosa*, or "true whitlow," is an advanced stage of the preceding, or arises—with no apparent injury to the surface—in the pulp of the fingers. *Staplylococci* are probably always present. The sheath of the tendon (usually of the flexors) is quickly affected, and extensive sloughing of the parts ensues owing to the great tension. For the same reason, and because of the numerous sensitive nerve endings present, the pain is very great.

One thing is necessary, viz., to make an early and deep incision in the middle line of the finger. This cut must be made down to the bone, or at least into the insertion of the tendon, and it is important that it should not be made quite at the finger end, but nearer the terminal joint. If the bone is necrosed (as is often the case) it should be well scraped with a Volkman's spoon, and the wound washed out with perchloride of mercury solution (1 in 1,000). Under this treatment very marked disease of the bone may be speedily cured, for the peristeum of the terminal phalanx has a very free supply of blood from the surrounding tissues, and this gives the bone great recuperative power.

On making the incision it is usual to find, in severe cases, grey-coloured granulations at the bottom of the cut; and if a probe be introduced it glides through these into the sheath of the tendon. These granulations often extend down the sheath for some distance, and in the case of the thumb and the little finger as far as the palm of the hand. Consequently, it is a frequent occurrence for amputations of the terminal phalanx for whitlow-necrosis to do badly and for a second amputation at the next joint to be necessary. This may be obviated by carefully examining the sheath and, if necessary, scraping it out with a fine Volkman's spoon or scoop-shaped director.

It is often necessary to give an anæsthetic in these cases. Cocaine is almost useless, and freezing by the ether spray is, with most people, the greatest agony. In strong people, however, the operation, if performed quickly, may be undertaken without producing general narcosis. Wrapping strong and hot solution of carbolic acid on lint round the finger for five minutes produces very considerable local anæsthesia.

As regards general treatment: pain, especially by depriving the patient of the night's rest, is the chief agent in increasing the ill-health. Consequently, opium or chloral are useful drugs, and should be administered at bedtime when the pain is very severe or an operation refused. A practical point is the arrangement of the arm, which should be supported from the finger to the elbow in a firm sling. For this purpose canvas or somewhat stiff material is better than yielding silk.

It is needless to point out that neglect of these cases on the part of the surgeon or the patient often leads to disastrous results, such as necrosis, contraction of the tendons, loss of power of the finger, anchylosis at the terminal point, severe impairment of health, and sometimes dangerous blood-poisoning.

We thus see that all the ordinary forms of paronychia have the same pathology and are merely stages of the same inflammatory affection. The treatment is equally clear.

The occupations that predispose to the disease (beside those when the fingers are constantly scratched and pricked) are chiefly (1) exposure to steam and hot water, such as occurs in laundry work; (2) working in alkali or acid works, or (3), certain chemicals, (4) varnishing, and the handling of irritating pigments, *e.g.*, chromates, &c.

No age is exempt, but it is commonest in middle-aged women.

